# A Comparison of the Effectiveness of Videolaryngoscopy and Macintosh Laryngoscopy in Intubation Attempts on Adult Patients

**DOI:** 10.5152/TJAR.2022.21367

**Published:** 2022-10-01

**Authors:** Haydar Bektaş, Sıtkı Göksu, Elzem Şen

**Affiliations:** 1 Department of Anaesthesiology and Reanimation, University of Gaziantep Faculty of Medicine, Gaziantep, Turkey

**Keywords:** Intubation, laryngoscopy, Macintosh blade, videolaryngoscopy

## Abstract

**Objective:**

This study aimed to compare the effectiveness of videolaryngoscopy and Macintosh laryngoscopy on adult patients who were scheduled for elective surgery under general anaesthesia.

**Methods:**

Of the 200 adult patients who were scheduled to undergo general anaesthesia, 100 were intubated with a videolaryngoscope and 100 with a Macintosh laryngoscope. The patients’ age, sex, American Society of Anesthesiologists score, height, weight, body mass index, smoking and alcohol habits, comorbidity, and neck circumference were recorded. Their El-Ganzouri Risk Index score, which considers the parameters of mouth opening, thyromental distance, Mallampati score, neck movement, propensity for prognathism, body weight, and history of difficult intubation, was also calculated and recorded. The time to achieve intubation was then recorded. The number of intubation attempts, number of cases of difficult intubation, Cormack–Lehane scores, and incidences of trauma or complication were also evaluated.

**Results:**

The mean intubation time was found to be significantly lower in the videolaryngoscope group compared to the Macintosh laryngoscope group. Although the number of patients with difficult intubation was high in the videolaryngoscope group, when we evaluated their glottic view, the Cormack–Lehane score was found to be significantly lower. The number and ratio of complications due to intubation were lower in the videolaryngoscope group compared to the Macintosh laryngoscopy group.

**Conclusions:**

In patients undergoing endotracheal intubation for general anaesthesia, it was concluded that videolaryngoscopy is superior to Macintosh laryngoscopy as it enlarges the glottic view, shortens the time to achieve intubation, facilitates intubation, and has less risk of complications.

**Main Points**

- Macintosh laryngoscope is the gold standart instrument for endotracheal entubation and most used worldwide in airway management.
- Use of videolaryngoscopy likely offers minimal benefits to patients with uncomplictad airway. It’s use is advantageous in these patients as training guide for practitioners.
- The videolaryngoscope therefore provides a clinically significant improvement in intubation conditions and is recommended for difficult airway management.
- Videolaryngoscope may improve safety by avoiding many unnecessary attempts.

## Introduction

Ensuring and managing airway safety is the first step in all anaesthetic procedures. Moreover, no anaesthetic is safe to administer unless a functional airway is established. Endotracheal intubation is frequently performed in anaesthetic procedures, especially in general anaesthesia. This method enables anaesthesiologists to keep the airway open, control breathing, adjust breathing effort, reduce dead space, prevent aspiration, ensure they themselves and their equipment are kept away from the surgical field, and manage airway during resuscitation. For these reasons, endotracheal intubation is the best method for ensuring and managing airway safety. In endotracheal intubation, a tube is placed into the trachea.^1^

For many years, a Macintosh laryngoscope blade has been used for this procedure. Recently, videolaryngoscopes have been increasingly used.^2^ It is thought that airway trauma is less likely to occur with a videolaryngoscope. In accordance with the difficult airway algorithm, the American Society of Anesthesiologists (ASA) recommends videolaryngoscopy as the first choice for all patients to be intubated.^3^

A larger and clearer image is obtained, compared to that achieved with a conventional laryngoscope, by a camera placed on the blade tip of the videolaryngoscope. This provides anaesthesiologists with the opportunity to better orient anatomical structures, increases the success of intubation, and is expected to shorten the time required to achieve intubation. As less force is applied, the risk of damage to the teeth or soft tissue trauma is also reduced.^4^

Laryngoscopy and endotracheal intubation often have undesirable effects, with an increase in heart rate, intravascular, intraocular, or intracranial pressure, as well as arrhythmia and bronchoconstriction frequently observed.^5^

Due to the fact that videolaryngoscopes are used to facilitate laryngoscopy and to improve the glottic view in difficult airways, they may exert less tension and lessen the severity of haemodynamic response.^6^ Numerous studies have been conducted and continue to be conducted on the advantages and disadvantages of videolaryngoscopes, and how intubation success compares with other methods. These studies are mostly studies aiming to compare the use of videolaryngoscopes with the classic Macintosh laryngoscope.

This present study aims to evaluate whether videolaryngoscopes are superior to the Macintosh blade in terms of their level of success in endotracheal intubation, time to achieve intubation, number of attempts, and complications.

## Methods

The study was planned prospectively upon receiving the approval of the Ethics Committee of Gaziantep University with the decision dated October 21, 2020, and numbered 2020/308. Written informed consent was obtained from patients who participated in this study. Two hundred patients between the ages of 18 and 65, who were scheduled to undergo elective surgery between October 1, 2020, and February 28, 2021, and would consequently be administered general anaesthesia were planned to be included in the study. Patients were randomised using a computer-generated randomisation program to the group to be intubated using a size 3 or 4 Macintosh laryngoscope blade (ML Group) or the group to be intubated using a videolaryngoscope (VL Group). Randomisation codes were put in closed opaque envelopes. The size of the laryngoscope used was left to the discretion of the anaesthesiologist conducting the procedure.

Patients with decompensated heart failure or elevated intracranial pressure and those who were in the ASA IV-V group, pregnant, or undergoing emergency surgery were excluded from the study.

The age, sex, ASA score, height, weight, body mass index (BMI), smoking and alcohol habits, comorbidity, El-Ganzouri Risk Index (EGRI) score, and neck circumference of the patients were recorded. The EGRI is a scoring index consisting of 7 parameters that is measured on a scale of 0-12[Table t1-tjar-50-5-352]. The parameters are mouth opening, thyromental distance, Mallampati score, neck movement, propensity for prognathism, body weight, and history of difficult intubation, and all were calculated and recorded for this index.

The EGRI is used as a bedside estimation score to increase patient safety before intubation by assessing the likelihood of a difficult airway.^7^ An EGRI score of 7 and above suggests that intubation may be difficult.^8-10^

The time to achieve intubation was then recorded. After intubation, the number of attempts and the incidence of difficult intubation, trauma, and complications were evaluated. Cormack–Lehane (CL) scoring was used to evaluate the glottic view. Each patient was administered 0.02 mg kg^−1^ of midazolam in the preoperative preparation room. The patients were administered 2 mg kg^−1^ of propofol IV, 1.5 µg kg^−1^ of fentanyl IV, and 0.6 mg kg^−1^ of rocuronium IV for induction of anaesthesia. They were then intubated after being ventilated with a mask for 3 minutes. The patients were administered 2% of sevoflurane and 0.1 µg kg^−1^ min^−1^ of remifentanil infusion for maintenance of anaesthesia.

Size 7, 7.5, and 8 tubes were used for intubation. The most important indicator of successful intubation is observing that the tube has passed between the vocal cords and entered the trachea. Also, an ETCO_2_ compatible with ventilation in capnography after 3 consecutive ventilator breaths indicates that the tube is not in the oesophagus. When this cannot be confirmed, that is, in grade III and IV laryngoscopies, the procedure is considered to be performed blindly and there is a 50% theoretical risk of oesophageal intubation. It is reported that most cases in which difficulties are encountered fall into grade III, whereas grade IV laryngoscopy is rare.^11^ Time to achieve intubation was regarded as the time elapsed between placing the laryngoscope in the patient’s mouth until 3 consecutive end-tidal curves were obtained in capnography.

The patient group intubated with a MEDCAPTAIN VS-10S videolaryngoscope was named the VL Group, and the patient group intubated with a Macintosh blade was named the ML Group. The blade types used on the VL Group were M3 and M3D. M3D was used more frequently on patients with a history of difficult intubation. In the ML Group, blades 3 and 4 were used. Patients who had previously had difficult intubation were prioritised for the videolaryngoscope group. In both groups, the intubation procedure was performed by inserting a stylet into the endotracheal tube (ETT) during intubation.

### Statistical Analysis

The conformity of numerical variables with normal distribution was analysed using the Shapiro–Wilk test. A Mann–Whitney *U* test was used in the comparison of abnormally distributed variables in 2 groups, and Kruskal–Wallis and Dunn’s tests were used in the comparison in more than 3 groups. Correlation between numerical variables was tested with the Spearman’s rank correlation coefficient test and correlation between categorical variables was tested with the chi-squared test. Windows version Statistical Package for the Social Sciences 22.0 package software (IBM Corp.; Armonk, NY, USA) was used in the analyses, and *P*  < .05 was regarded as significant.

## Results

Demographic data are shown in Table 1 for both groups. When the demographic data were compared between the 2 groups, no statistically significant difference was found. However, a significant difference was observed between times to achieve intubation.

**Figure 1.**
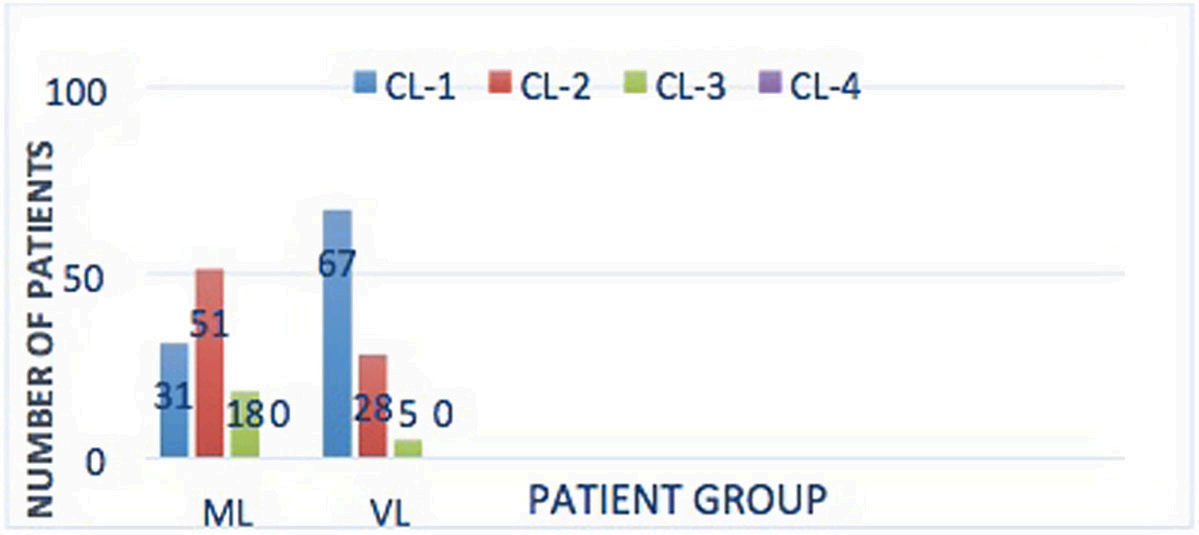
Cormack-Lehane scoring information between groups. CL, Cormack-Lehane score; ML, Macintosh laryngoscope; VL, videolarynoscope.

Cormack–Lehane scoring information for the VL Group and ML Group is given in Figure 1. The number of patients with Cormack–Lehane Score I (CL-I) in the VL Group was 67, while 28 were CL-II, and 5 were CL-III. The number of patients with CL-I in the ML Group was 31, while 51 were CL-II, and 18 were CL-III. There were no CL-IV patients in either group (*P*  = .001).

**Figure 2.**
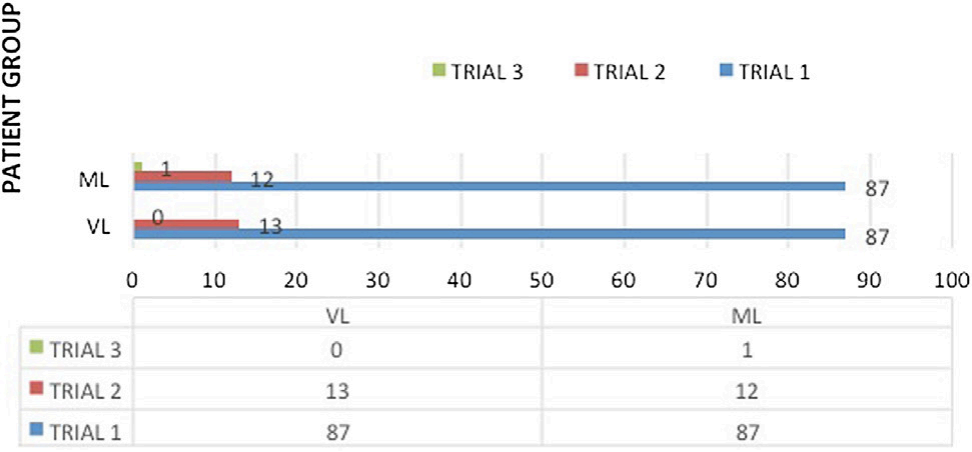
Number of intubation attempts for patients in both groups. ML, Macintosh laryngoscope; VL, videolaryngoscope.

The number of intubation attempts for the patients in the VL Group and ML Group is given in Figure 2. In the VL Group, the number of patients needing 1 intubation attempt was 87, while 13 required 2 attempts, and no patients needed 3 attempts. In the ML Group, the number of patients needing 1 intubation attempt was 87, 12 required 2 attempts, and 1 underwent 3 attempts. The numbers of intubation attempts required were not statistically significant.

The EGRI Index information for the patients in both groups is given in Table 2. There was a statistically significant difference in the number of patients with difficult intubation in the VL Group, as patients with suspected and existing difficult intubation were prioritised for videolaryngoscopy. No statistically significant difference was observed between EGRI scores.

Information on intubation-related complications for the VL Group and the ML Group is given in Table 3. In the VL Group, the number of patients with no intubation-related complications was 92 (92%), the number of patients with blood in the oropharynx was 2 (2%), the number of patients with blood on the laryngoscope was 1 (1%), the number of patients with pharynx-larynx and intraoral mucosal damage was 3 (3%), and the number of patients with oesophageal intubation was 2 (2%). In the ML Group, the number of patients with no intubation-related complications was 81 (81%), the number of patients with blood in the oropharynx was 0, the number of patients with blood on the laryngoscope was 8 (8%), the number of patients with pharynx-larynx and intraoral mucosal damage was 6 (6%), and the number of patients with oesophageal intubation was 5 (5%). The *P* value was statistically significant between these 2 groups (*P*  < .017).

## Discussion

Videolaryngoscopy improves intubation success by improving the glottic view in cases where seeing the glottis is difficult.^11^ During direct laryngoscopy, maneuvers such as the “sniffing” position and external movement of the larynx with cricoid pressure are used to improve the field of view.^12^

More rapid and reliable endotracheal intubation is recommended in patients with a high risk of spreading an aerosolised virus, such as coronavirus-2019 infection. If possible, intubation should be carried out with a videolaryngoscope. This is because the prolongation of time to achieve intubation and an increased number of attempts also increase exposure to the aerosolised virus.^13^

Numerous randomised, controlled studies have been conducted comparing videolaryngoscopy with direct laryngoscopy in patients predicted to have a difficult airway. In different meta-analyses drawing from these studies, when compared to direct laryngoscopy, videolaryngoscopy has been shown to provide a clearer view of the larynx, increase the frequency of successful intubation, and increase the frequency of successful intubation on the first attempt.^14-16^

Kaur et al^17^ compared the effectiveness of a McGrath MAC videolaryngoscope, a Truview videolaryngoscope, and a Macintosh laryngoscope on endotracheal intubation in patients who were to be operated on under general anaesthesia. Time to achieve intubation was shorter in both videolaryngoscope groups. In videolaryngoscope group only CL I and II views were seen, but in Machintosh laryngoscope group CL III and IV views were seen additionally. There were 2 complications in videolaryngoscope group and 5 complications in Machintosh laryngoscope group. Better glottic view and fewer complications were seen for the patients in the videolaryngoscope groups.^[Bibr b17-tjar-50-5-352]^

In their prospective study, Abdallah et al^18^ found that the Airtraq videolaryngoscope ensured easier intubation than Macintosh laryngoscopy. Average time to achieve intubation was 14.18 seconds in the Macintosh laryngoscope group, and 11.5 seconds in the videolaryngoscope group. They found that a videolaryngoscope facilitates intubation and causes complications less frequently.

Reena et al^19^ compared patients on whom a KingVision videolaryngoscope (non-channelled) and a Macintosh laryngoscope were used for endotracheal intubation with an armoured ETT. The videolaryngoscope was determined to be superior in time to achieve intubation and first attempt success.

Zhu et al^20^ compared a KingVision videolaryngoscope (non-channelled) with a McGrath MAC videolaryngoscope, and a Macintosh laryngoscope in patients with difficult intubation requiring nasotracheal intubation. They demonstrated that videolaryngoscope groups were seen to have a higher percentage of first intubation success, a better glottic view, and a lower incidence of complications.

Cavus et al^21^ evaluated videolaryngoscopy in both normal and difficult intubations and concluded that a videolaryngoscope increases the success of endotracheal intubation in patients for whom a difficult airway is both expected or not by providing a better glottic view.

Hoshijima et al^22^ conducted a systematic review and meta-analysis of 18 randomised, controlled trials to compare the C-MAC videolaryngoscope with the Macintosh laryngoscope for tracheal intubation in the adult population and, as a result, showed that the videolaryngoscope offered a better glottic view and required less external laryngeal manipulation compared to the Macintosh laryngoscope.^[Bibr b22-tjar-50-5-352]^

Serocki et al^23^ compared an unnamed videolaryngoscope and a GlideScope with a Macintosh blade in terms of glottic view and intubation success in 96 patients who were due to undergo ENT surgery and had suspected difficult intubation. They concluded that both videolaryngoscopes improved the glottic view and were useful alternatives for the management of difficult airway.

In a randomised meta-analysis study, Su et al^11^ compared videolaryngoscopy with direct laryngoscope in which 1196 patients and 11 teams took part. They found that videolaryngoscopy was far superior in terms of glottic view and its intubation success was better. They also showed that time to achieve intubation was shortened in patients with difficult intubation.

Liu et al^24^ compared videolaryngoscopy and direct laryngoscopy in endotracheal intubation in non-difficult airways in which 360 patients were included. The percentage of patients with a level I-II total glottic exposure in the videolaryngoscope group was 100%, while it was 63.5% in the direct laryngoscope group. The single attempt success rate of intubation was seen to be 96.1% in the videolaryngoscopy group and 90.1% in the direct laryngoscopy group.

As the technology develops, improvements in videolaryngoscopes make it easier to use and provide a more easily obtainable and clearer glottic view. This facilitation leads to a shortening of time to achieve intubation and a reduction in intubation-related complications. The videolaryngoscope therefore provides a clinically significant improvement in intubation conditions and is recommended for difficult airway management. Despite being usually used in cases where difficult intubation is expected, videolaryngoscopy can also be used in all cases requiring tracheal intubation.

## Conclusion

In our study, the glottic view was better for the cases in the videolaryngoscope group. This group had a reduced time to achieve intubation and intubation was also facilitated, causing less trauma. In order to reduce complications in cases where tracheal intubation is required, especially in unpredictable, difficult airways, videolaryngoscopy is therefore recommended.

## Figures and Tables

**Table 1. t1-tjar-50-5-352:** Demographic Data

**Parameter**	**ML Group (*n* = 100)**	**VL Group (*n* = 100)**	***P***
Age (years) (mean ± SD)	42.80 ± 14.52	42.73 ± 14.36	.947
BMI (kg cm–^2^) (mean ± SD)	28.10 ± 5.23	28.12 ± 5.57	.880
Neck circumference (cm) (mean ± SD)	37.43 ± 4.30	37.39 ± 4.44	.943
Sex (F/M) (n) (%)	61 (61%)/39 (39%)	60 (60%)/40 (40%)	.885
Smoker (no/yes) (n)	65/35	68/32	.653
Alcohol consumer (no/yes) (n)	86/14	87/13	.836
Comorbidity (no/yes) (n)	56/44	57/43	.887
EGRI score (mean ± SD)	2.61 ± 1.89	2.89 ± 2.11	.488
Intubation time (seconds) (mean ± SD)	34.01 ± 22.08	26.09 ± 15.70	.001*
ASA classification			
I-(n)	I-36	I-42	.644
II-(n)	II-44	II-36	
III-(n)	III-20	III-22

*P  < .05

ML, Macintosh laryngoscope; VL, videolaryngoscope; SD, standard deviation; BMI, body mass index; F, female; M, male; EGRI, El-Ganzouri Risk Index; ASA, American Society of Anesthesiologists.

**Table 2.** El-Ganzouri Risk Index Scores for Both Groups

**Variable**		**ML Group (*n* = 100)**		**VL Group (*n* = 100)**		***P***
**(n)**	**(%)**	**(n)**	**(%)**
Mouth opening (cm)	≥4	83	83	81	81	.713
<4	17	17	19	19
Thyromental distance (cm)	>6.5	81	81	85	85	.751
	6-6.5	15	15	12	15	
<6.5	4	4	3	3
Mallampati score	M1	21	21	17	17	.421
	M2	36	36	45	45	
M3	43	43	38	38
Body weight (kg)	<90	92	92	87	87	
	90-110	8	8	10	10	
110<	0	0	3	3
Maximal neck movement (°)	>90	46	46	51	51	.619
	80-90	53	53	47	47	
<80	1	1	2	2
Propensity for prognathism	Definite	71	71	59	59	0.075
None	29	29	41	41
History of difficult intubation	None	91	91	84	84	.044*
	Questionable	9	9	12	12	
Definite	0	0	4	4
EGRI	None≤7	100	100	98	98	.095
Yes>7	0	0	2	2

*P  < .05.

EGRI, El-Ganzouri Risk Index; ML, Macintosh laryngoscope; VL, videolaryngoscope.

**Table 3.** Information of Complications Related to Intubation in the ML Group and VL Group

**Intubation-Related Complications**	**ML Group (*n* = 100)**	**VL Group (*n* = 100)**	***P***
**Yes** ** Blood in the oropharynx (n) (%)** ** Blood on the laryngoscope (n) (%)** ** Pharynx, larynx, and intraoral** ** mucosal damage (n) (%)** ** Oesophageal intubation (n) (%)**	0 (0)	2 (2)	.017*
	8 (8)	1 (1)	
	6 (6)	3 (3)	
5 (5)	2 (2)		
**None (n) (%)**	81 (81)	92 (92)

* *P*  < .05.

ML, Macintosh laryngoscope; VL, videolaryngoscope.
